# Analysis of tuberculosis treatment outcomes among pulmonary tuberculosis patients in Bahawalpur, Pakistan

**DOI:** 10.1186/s13104-018-3473-8

**Published:** 2018-06-08

**Authors:** Muhammad Atif, Zainab Anwar, Razia Kaneez Fatima, Iram Malik, Saima Asghar, Shane Scahill

**Affiliations:** 1 Department of Pharmacy, The Islamia University of Bahawalpur, Bahawalpur, Pakistan; 2 National Tuberculosis Control Program of Pakistan, Islamabad, Pakistan; 3 Massey University, Auckland, New Zealand

**Keywords:** Pulmonary tuberculosis, New smear positive pulmonary tuberculosis, Treatment outcomes, Unsuccessful treatment outcome, Pakistan, High TB burden countries

## Abstract

**Objective:**

Monitoring tuberculosis treatment outcomes and understanding the reasons for unsuccessful treatment are important indicators for evaluating the performance of the national tuberculosis control program. The aim of this study was to evaluate the treatment outcomes among pulmonary TB (PTB) patients and identify the predictors of unsuccessful treatment outcome.

**Results:**

Treatment success rate of 67.8% among new and retreatment PTB patients and 69% in new smear positive PTB patients was observed. Close to 21% (20.9%) and 15.7% PTB and new smear positive PTB patients had loss to follow-up during treatment. Overall, older patients (AOR 1.02; 95% CI 1.01–1.0), smokers (AOR 1.65; 95% CI 1.02–2.67) and retreatment cases of TB (AOR 2.34; 95% CI 1.43–3.84) were at greater risk of having unsuccessful treatment outcomes. Moreover, sputum positivity at 2 months (AOR 13.78; 95% CI 5.09–37.26) was a significant predictor of poor treatment outcomes in new smear positive PTB patients. The treatment success rate among PTB patients was lower than the recommended 85% success rate. TB patients at higher risk of unsuccessful treatment outcomes should be provided with enhanced supervision and treatment monitoring to improve the success rate of TB management in Pakistan.

**Electronic supplementary material:**

The online version of this article (10.1186/s13104-018-3473-8) contains supplementary material, which is available to authorized users.

## Introduction

Despite the fact that tuberculosis (TB) is a preventable disease, it still ranks among the top ten causes of death worldwide [1]. According to a recent report by the World Health Organization (WHO) in 2016, around 10. million people were infected with *Mycobacterium tuberculosis* and 1.7 million people died (including .4 million deaths among human immunodeficiency virus (HIV)-positive people) due to TB [2]. According to 2016 estimates, 56% of people suffering from TB were living in five countries (in descending order); India, Indonesia, China, the Philippines and Pakistan [1]. Pakistan shares 61% of the TB burden in the WHO Eastern Mediterranean Region [3]. In 2016, 356,390 new and relapsed cases of TB were notified in Pakistan, showing an increase in the number of notified cases compared with 2015 (323,856 cases) [1, 4]. Among all notified cases in 2016, 80% were pulmonary tuberculosis (PTB) cases, and 4% cases had known HIV infection [1]. Although trends in TB mortality rates in Pakistan from 2012 to 2016 demonstrate a substantial decline in associated deaths ranging from 34 to 23 cases per 100,000 population [1, 4–7], TB remains a significant killer in this country.

To control the global burden of TB, in 2006 the WHO developed the Stop TB Strategy (2006–2015) that was built on the Stop TB Partnership’s first global plan (2001–2005) [8]. The main targets outlined in the strategy linked to the Millennium Development Goals (MDGs) were reduction in TB prevalence and mortality rate due to TB by 50% by 2015 as compared to 1990 [6]. Pakistan has met this Stop TB target of 50% reduction in TB mortality rates over the specified time and has made enormous progress regarding successful treatment of drug-susceptible TB. However, the country still faces a burdensome prevalence rate of 341 cases per 100,000 population and an incidence rate of 270 cases per 100,000 [4, 6]. Moreover, in recent years, Pakistan is estimated to stand fourth among countries of the world with the highest proportion of multidrug-resistant TB (MDR-TB) [9].

The WHO recommends that treatment outcome analysis among PTB patients be carried out every year at national and district levels [10]. Considering the recommendations of the WHO and taking into account the steady surge in burden of TB in Pakistan, this study aims to assess treatment outcomes of PTB patients including newly diagnosed smear positive PTB cases at the Bahawal Victoria Hospital (BVH) in southern Punjab, Pakistan.

## Main text

### Methods

#### Study setting

This study was undertaken at the TB DOTS (directly observed treatment, short course) clinic in the Respiratory Department of the BVH. The BVH, a 1600 bedded health facility, serves as a tertiary care referral hospital in the southern region of Punjab, Pakistan. The Chest Disease Unit (CDU) of the BVH has 8–10 physicians, 5–6 chest specialists and two pharmacists who provide routine care to patients with chest-related diseases [11]. The TB outdoor clinic is visited by 35–40 TB patients daily. The TB unit within the chest clinic works under the National Tuberculosis Control Program (NTP) [11].

As suggested by the NTP guidelines, ‘presumptive TB cases’ are identified at the TB outdoor clinic based on their symptoms and follow-up microscopy [12]. The TB DOTS clinic has a separate diagnostic laboratory and an X-ray room. Smear positive patients are referred for registration, and TB treatment is provided free of charge to the patient.

#### Study design and data collection

This was a retrospective study including all new and retreatment smear negative and smear positive PTB patients (1 year cohort) diagnosed and registered at BVH between January 1 and December 31, 2014. Medical registers and TB treatment cards were reviewed to collect socio-demographic, clinical and treatment-related data [13, 14]. Individual treatment files were also reviewed to obtain biochemical and hematological data of PTB patients.

#### Reporting of treatment outcomes

The outcomes of treatment were reported in accordance with six outcome categories developed and recommended by the WHO and the International Union Against Tuberculosis and Lung Disease (IUATLD). This allows standardized reporting of treatment outcomes among all new and retreatment PTB patients [10]. Descriptions for outcome categories are provided in Additional file 1: Table S1.

#### Statistical analysis

Data were analyzed using the Statistical Package for Social Sciences (IBM, SPSS Statistics for Windows, version 21.0. Armonk, NY: IBM Corp.). Categorical variables were analyzed using counts and proportions (%). Continuous variables were described in terms of mean and standard deviations (SD). Simple logistic regression analysis was applied to evaluate the relationship between the dependent variable (i.e., unsuccessful treatment outcome) and the selected socio-demographic and clinical variables. Statistically significant variables in univariate analysis were analyzed using multiple logistic regression analysis to determine the final predictors of unsuccessful treatment outcome. The beta, standard error, adjusted odd ratios (AOR), 95% CI and *p* value were documented for each predictor [15].

### Results

A total of 969 TB patients were registered at the study site. Out of these, 690 (71.2%) PTB patients were included in the study. Over one quarter of patients (279, 28.8%) were excluded with 270 (27.8%) patients having EPTB, 8 (.8%) having incomplete records of TB treatment and 1 (.1%) being diagnosed with MDR-TB. In terms of microscopic findings, 283 (41%) patients were smear positive while the remaining 407 (59%) were smear negative. Among the 283 smear positive cases, the large majority (242, 85.5%) were new cases of PTB. For details, please refer to Table 1.

**Table 1:** Socio-demographic characteristics, clinical characteristics and resource utilization of new and retreatment pulmonary tuberculosis patients (n = 690)

Patient characteristics	Patients n (%)
Sex	
Male	357 (51.7)
Female	333 (48.3)
Age group, mean age 36 (SD 8.98) years	
0–14	29 (4.2)
15–24	230 (33.3)
25–34	116 (16.8)
35–44	84 (12.2)
45–54	80 (11.6)
55–64	70 (10.1)
≥ 65	81 (11.7)
Distance from the treatment center (km)	
≤ 5	316 (45.8)
6–25	280 (40.6)
26–50	48 (7.0)
> 50	46 (6.7)
Smoking status	
Non-smoker	597 (86.5)
Smoker	93 (13.5)

Clinical characteristics	Patients n (%)
Form of PTB	
S^**+**^ PTB*	283 (41.0)
New patient	242 (85.5)
Retreatment patient	41 (14.5)
S^−^ PTB**	407 (59.0)
New patient	369 (90.7)
Retreatment patient	38 (9.3)
Type of patient	
New cases	611 (88.6)
Retreatment cases	79 (11.4)
Baseline weight (kg)	
< 47	493 (71.4)
≥ 47	197 (28.6)
Comorbidities	
Diabetes	47 (6.8)
Hypertension	39 (5.6)
Hepatitis	3 (.43)
Bacteriological tests performed	
Sputum smear examination	690 (100.0)

* Smear positive pulmonary tuberculosis

** Smear negative pulmonary tuberculosis

Out of 690 PTB patients, only 468 (67.8%) patients were found to be successfully treated. Among the unsuccessfully treated patients, 14 (2.0%) failed the treatment, 144 (20.9%) were lost to follow-up and 35 (5.1%) died during the treatment course (Additional file 2: Table S2). Of all new smear positive PTB patients, 163 (67.3%) were reported as cured and 4 (1.7%) completed the treatment (Table 2).

**Table 2:** Treatment outcomes of new smear positive pulmonary tuberculosis patients as per the Standard criteria (n = 242)

Treatment outcomes	Patients n (%)	Total n (%)
Successful		
Cured	163 (67.3)	167 (69.0)
Treatment completed	4 (1.7)	
Unsuccessful		
Treatment failure	10 (4.1)	
Loss to follow-up	38 (15.7)	75 (31.0)
Died	18 (7.4)	
Not evaluated	9 (3.8)	

World Health Organization and International Union Against Tuberculosis and Lung Disease Criteria

Through multiple logistic regression analysis, factors that remained significantly associated with the unsuccessful treatment outcome among new and retreatment PTB patients were; age 45 years and above (OR 1.02; 95% CI 1.01, 1.03, p < .0005), being a retreatment case of PTB (OR 2.34, 95% CI 1.43, 3.84, p = .001) and being a smoker (OR 1.65, 95% CI 1.02, 2.67, p = .04). For the new smear positive PTB patients, the only determinant in the multiple logistic regression analysis which was significantly related to the unsuccessful treatment outcome was sputum positivity at 2 months (OR 13.78; 95% CI 5.09, 37.26, p < .0005) (Table 3).

**Table 3:** Predictors of unsuccessful treatment outcome: Multiple logistic regression analysis

Independent variables	*B*	*S.E*	*p value*	AOR (95% CI)
Among all PTB patients*				
Older age	.02	.00	*< .0005*	1.02 (1.01, 1.0)
Retreatment case	.85	.25	*.001*	2.34 (1.43, 3.84)
S^+^ PTB	.17	.17	.323	1.18 (.84, 1.67)
Hypertension	.13	.35	.711	1.14 (.56, 2.30)
Diabetes	.25	.33	.452	1.28 (.66, 2.47)
Smoker	.50	.24	*.040*	1.65 (1.02, 2.67)
Among new smear positive PTB patients^†^				
Age	.14	.49	.782	1.15 (.44, 3.01)
Sputum positivity at 2 months	2.62	.51	*< .0005*	13.78 (5.09, 37.26)
Smoker	1.06	.60	.079	2.89 (.88, 9.43)

Italic values indicate statistically significant (*p* < 0.05)

*Model summary (Chi square = 58.870, degrees of freedom = 6, p < .0005, pseudo R2 = .114)

^†^Model summary (Chi square = 34.199, degrees of freedom = 3, p < .0005, pseudo R2 = .291)

### Discussion

In this study, the overall treatment success rate among PTB patients was low. In addition, treatment success in new smear positive PTB patients was less than the WHO successful target rate of 85%. Along similar lines, other high TB burden countries such as Somalia (81.8%) [16], Nigeria [17] India (74%) and Brazil (71%) have reported suboptimal treatment success rates [4]. Interestingly, in contrast to our findings, the overall treatment success rate in the Eastern Mediterranean Region of the WHO including Pakistan was above 90% for the 2014 cohort [4]. This variation in TB treatment outcomes around the world might be explained by different factors such as the study design and sample size of study population, complexity of the disease among the patients, quality of facilities provided at the treatment center, HIV status of patients and local beliefs among TB patients about the DOTS strategy.

In the current study the major reasons for the lower treatment success rate were firstly, higher loss to follow-up and secondly, the death rate. Studies from Libya and Ethiopia also documented consistent findings regarding loss to follow-up among smear positive PTB patients [14, 18]. At the local level, patient loss to follow-up might also be compounded by the need to purchase medicines to alleviate the side effects of TB medicines in those who already have poor financial status. As reported in the literature [19], patients’ time constraints, long distances from TB clinics and stigmatizing attitudes towards their illness might contributed towards treatment non-compliance. The second major factor responsible for lower treatment success rates was the higher death rate amongst our Pakistani patients. This is in agreement with previously conducted studies in Singapore and the United States [20, 21]. A higher death rate in our study could be attributed to the study setting. BVH is a tertiary care referral hospital in the southern region of Punjab, Pakistan and it is likely that a relatively higher number of complex TB patients are registered or referred to the BVH, thereby contributing towards the high mortality rate. Moreover, the higher loss to follow-up rate might have contributed to the development of a more complex and drug resistant M. TB strain among the TB patients. This would result in an increase in the risk of death.

In the multiple logistic regression analysis, being a retreatment case, older age, and being a smoker were the independent predictors of unsuccessful treatment outcomes among PTB patients. In line with our findings, studies conducted elsewhere have reported poor treatment outcomes among retreatment TB cases [14, 17, 22]. Similarly, the current study revealed that there is relatively higher treatment failure rates amongst retreatment cases (Additional file 3: Table S3) and that could be a consequence of possible MDR-TB in this subgroup. Unfortunately, at the time the study was conducted the WHO recommended [10] culture and drug susceptibility testing (DST) that were not accessible for complex TB cases at BVH (now these services are available).

This study highlighted that older patients were at greater risk of having poor treatment outcomes for TB. Similar to our findings, a previous Pakistani study [23] and an Ethiopian study [14] reported increased age to be associated with unsuccessful treatment outcome. As older patients are reasonably fragile, non-ambulatory and reliant on family members for transport to health centers for their ongoing treatment there are more likely to be treatment interruptions in this cohort. Smoking was another independent risk factor associated with the unsuccessful treatment outcome among PTB patients. In line with our findings, a recently conducted study in Pakistani TB patients found smoking to be significantly associated with unsuccessful treatment outcomes [23]. Similarly, other studies showed higher unsuccessful treatment outcomes possibly due to treatment failure among TB patients who smoke [24].

Analysis of treatment outcomes among new smear positive PTB patients is important to conduct, as they are major indicators of NTP performance. In this study, along with the assessment of treatment outcomes in new smear positive PTB patients, risk factors for unsuccessful treatment outcomes were also determined. Our study revealed sputum positivity at 2 months to be an independent predictor of unsuccessful treatment outcome among new smear positive PTB patients. All 10 patients in our study with a positive sputum smear result at 2 months failed treatment, indicating direct association of sputum positivity at 2 months with treatment failure among smear positive PTB patients. Similar to our findings, studies from India and South Africa have reported significant associations between sputum non-conversion and poor treatment outcomes [25, 26].

In conclusion, the treatment success rate among PTB patients in this Pakistani study were lower than the expected success target of 85%. Similarly, treatment success rates in new smear positive PTB cases were also less than targets set by the WHO. A large proportion of patients were lost to follow-up and died during treatment, which causes serious concern and warrants urgent action. Effective tracing methods for patients lost to follow-up should be developed and implemented to minimize treatment interruptions. Moreover, patients with an increased risk of having unsuccessful treatment outcomes should be provided with enhanced supervision and treatment monitoring to improve outcomes.

## Limitations

Our study as a few limitations. First, the findings cannot be generalized to indicate TB treatment success rate in the whole of Pakistan. This is because, BVH serves a complex population of TB patients from distant and rural areas. Indeed, the higher proportion of loss to follow-up and the death rate support this notion. Second, the retrospective design of this study is another limitation. It was not possible to prospectively access all of the patients’ clinical variables, information regarding major side effects of anti-TB drugs and factors that might have affected loss to follow-up and death rates (these were not written in patient charts).

## Additional files

**Additional file 1: Table S1.** Definition of treatment outcomes.

**Additional file 2: Table S2.** Treatment outcomes of all smear negative and smear positive pulmonary tuberculosis patients as per the Standard* Criteria (n = 690).

**Additional file 3: Table S3.** Number of new and retreatment smear negative and smear positive pulmonary tuberculosis patients by treatment outcomes category (n = 690).

## Abbreviations

AOR: adjusted odds ratios
BVH: Bahawal Victoria Hospital
CDU: Chest Disease Unit
DOTS: directly observed treatment, short course
DST: drug sensitivity testing
HIV: human immunodeficiency virus
IUATLD: International Union Against Tuberculosis and Lung Disease
MDR-TB: multidrug resistant tuberculosis
MGDs: Millennium Development Goals
NTP: National Tuberculosis Control Program
PTB: pulmonary tuberculosis
SD: standard deviation
TB: tuberculosis
WHO: World Health Organization
