# Supplementary material for: Analysis of tuberculosis treatment outcomes among pulmonary tuberculosis patients in Bahawalpur, Pakistan
Source: BMC Res Notes. 2018 Jun 8;11:370. doi: 10.1186/s13104-018-3473-8 (PMC5994136; doi:10.1186/s13104-018-3473-8)
Supplement: Supplementary file 1 — Additional file 1: Table S1. Definition of treatment outcomes. [file 13104_2018_3473_MOESM1_ESM.docx]

**Additional File 1**

**Table S1: Definition of Treatment Outcomes**

| **Outcome** | **Definition** |
| --- | --- |
| Cured | A patient with bacteriologically confirmed TB at the beginning of treatment who was smear- or culture-negative in the last month of treatment and on at least one previous occasion. |
| Treatment completed | A TB patient who completed treatment without evidence of failure but with no record to show that sputum smear or culture results in the last month of treatment and on at least one previous occasion were negative, either because tests were not done or because results are unavailable. |
| Treatment failed | A TB patient whose sputum smear or culture is positive at month 5 or later during the treatment. |
| Died | A TB patient who dies for any reason before starting or during the course of treatment. |
| Loss to follow-up | A TB patient who did not start treatment or whose treatment was interrupted for 2 consecutive months or more. |
| Not evaluated | A TB patient for whom no treatment outcome is assigned. This includes cases “transferred out” to another treatment unit as well as cases for whom the treatment outcome is unknown to the reporting unit. |

*Notes:* Adapted from Definitions and Reporting Framework for Tuberculosis-2013 revision, World Health Organization, 2013, Geneva, Switzerland
