# Supplementary material for: Analysis of tuberculosis treatment outcomes among pulmonary tuberculosis patients in Bahawalpur, Pakistan
Source: BMC Res Notes. 2018 Jun 8;11:370. doi: 10.1186/s13104-018-3473-8 (PMC5994136; doi:10.1186/s13104-018-3473-8)
Supplement: Supplementary file 2 — Additional file 2: Table S2. Treatment outcomes of all smear negative and smear positive pulmonary tuberculosis patients as per the Standard* Criteria (n = 690). [file 13104_2018_3473_MOESM2_ESM.docx]

**Additional File 2**

**Table S2: Treatment outcomes of all smear negative and smear positive pulmonary tuberculosis patients as per the Standard* Criteria (n = 690)**

| **Treatment outcomes** | **New**  **(S^−^ and S^+^ PTB**) patients**  **(n = 611)**  **n (%)** | **Retreatment**  **(S^−^ and S^+^ PTB**) patients**  **(n = 79)**  **n (%)** | **Total**  **patients**  **(n = 690)**  **n (%)** | **Total**  **n (%)** |
| --- | --- | --- | --- | --- |
| **Successful***** | 429 (70.2) | 39 (49.3) | 468 (67.8) | 468 (67.8) |
| **Unsuccessful** |  |  |  |  |
| Treatment failure | 11 (1.8) | 3 (3.8) | 14 (2.0) | 222 (32.2) |
| Defaulter | 119 (19.5) | 25 (31.6) | 144 (20.9) |  |
| Died | 31 (5.0) | 4 (5.1) | 35 (5.1) |  |
| Not evaluated | 21 (3.4) | 8 (10.1) | 29 (4.2) |  |

*World Health Organization and International Union Against Tuberculosis and Lungs Disease Criteria; **Smear negative and smear positive pulmonary tuberculosis; ***As S^−^ PTB cannot be classified as cured, therefore a broader category “Successful outcome” was used
