# Supplementary material for: Analysis of tuberculosis treatment outcomes among pulmonary tuberculosis patients in Bahawalpur, Pakistan
Source: BMC Res Notes. 2018 Jun 8;11:370. doi: 10.1186/s13104-018-3473-8 (PMC5994136; doi:10.1186/s13104-018-3473-8)
Supplement: Supplementary file 3 — Additional file 3: Table S3. Number of new and retreatment smear negative and smear positive pulmonary tuberculosis patients by treatment outcomes category (n = 690). [file 13104_2018_3473_MOESM3_ESM.docx]

**Additional File 3**

**Table S3: Number of new and retreatment smear negative and smear positive pulmonary tuberculosis patients by treatment outcomes category (n = 690)**

| **Patient**  **characteristics** | **Successful Outcome** | | **Success**  **rate (%)** | **Unsuccessful Outcome** | | | | **Total**  **patients**  **n** |
| --- | --- | --- | --- | --- | --- | --- | --- | --- |
|  | **Cured**  **n** | **Treatment completed**  **n** |  | **Treatment**  **failure**  **n** | **Default**  **n** | **Died**  **n** | **Not**  **evaluated**  **n** |  |
| **Sex** |  |  |  |  |  |  |  |  |
| Male | 92 | 143 | 65.8 | 7 | 77 | 21 | 17 | 357 |
| Female | 87 | 146 | 70.0 | 7 | 67 | 14 | 12 | 333 |
| **Age group (years)** |  |  |  |  |  |  |  |  |
| 0−14 | 2 | 21 | 79.3 | 1 | 5 | 0 | 0 | 29 |
| 15−24 | 57 | 121 | 77.4 | 1 | 44 | 1 | 6 | 230 |
| 25−34 | 37 | 51 | 75.9 | 2 | 20 | 0 | 6 | 116 |
| 35−44 | 27 | 23 | 59.5 | 1 | 26 | 2 | 5 | 84 |
| 45−54 | 23 | 32 | 68.7 | 3 | 16 | 1 | 5 | 80 |
| 55−64 | 21 | 20 | 58.6 | 3 | 15 | 9 | 2 | 70 |
| ≥ 65 | 12 | 21 | 40.7 | 3 | 18 | 22 | 5 | 81 |
| **Distance** |  |  |  |  |  |  |  |  |
| ≤ 5km | 84 | 134 | 69.0 | 6 | 62 | 17 | 13 | 316 |
| > 5km | 95 | 155 | 66.8 | 8 | 82 | 18 | 16 | 374 |
| **Type of patient** |  |  |  |  |  |  |  |  |
| New patients | 163 | 266 | 70.2 | 11 | 119 | 31 | 21 | 611 |
| Retreatment patients | 16 | 23 | 49.4 | 3 | 25 | 4 | 8 | 79 |
| **Form of PTB** |  |  |  |  |  |  |  |  |
| S^+^ PTB* | 177 | 2 | 63.2 | 14 | 54 | 19 | 17 | 283 |
| S^−^ PTB** | − | 289 | 71.0 | − | 90 | 16 | 12 | 407 |
| **Baseline weight (kg)** |  |  |  |  |  |  |  |  |
| < 47 | 128 | 195 | 323 | 10 | 108 | 30 | 22 | 493 |
| ≥ 47 | 51 | 94 | 145 | 4 | 36 | 5 | 7 | 197 |
| **High grade sputum** |  |  |  |  |  |  |  |  |
| No | 140 | 0 | 62.8 | 7 | 45 | 17 | 14 | 223 |
| Yes | 37 | 2 | 65.0 | 7 | 9 | 2 | 3 | 60 |
| **Diabetic** |  |  |  |  |  |  |  |  |
| No | 168 | 275 | 68.9 | 14 | 130 | 28 | 28 | 643 |
| Yes | 11 | 14 | 53.2 | 0 | 14 | 7 | 1 | 47 |
| **Hypertension** |  |  |  |  |  |  |  |  |
| No | 169 | 279 | 68.8 | 11 | 136 | 29 | 26 | 651 |
| Yes | 10 | 10 | 51.3 | 3 | 8 | 6 | 2 | 39 |
| **Smoker** |  |  |  |  |  |  |  |  |
| No | 164 | 255 | 70.2 | 11 | 120 | 24 | 23 | 597 |
| Yes | 15 | 34 | 52.7 | 3 | 24 | 11 | 6 | 93 |

*Smear Positive Pulmonary Tuberculosis; **Smear Negative Pulmonary Tuberculosis
